# Women's Intention to Participate in Predictive Breast Cancer Genetic Testing: A Cross‐Sectional Survey of in Iran

**DOI:** 10.1002/hsr2.70985

**Published:** 2025-07-09

**Authors:** Zahra Meshkani, Soheila Damiri, Najmeh Moradi, Mohadeseh Motamed‐Jahromi, Ali Aboutorabi

**Affiliations:** ^1^ National Center for Health Insurance Research Tehran Tehran Province Iran; ^2^ Health Management and Economics Research Center, Health Management Research Institute Iran University of Medical Sciences Tehran Tehran Province Iran; ^3^ Department of Health Management, Policy and Economics, School of Public Health Tehran University of Medical Sciences Tehran Tehran Province Iran; ^4^ Population Health Sciences Institute, Newcastle University Newcastle upon Tyne England U.K; ^5^ Department of Public Health, School of Health Fasa University of Medical Sciences Fasa Fars Province Iran

**Keywords:** BRCA1/BRCA2 variants, breast cancer genetic testing, health behavior intention, Iranian women's health, predictive genetic screening

## Abstract

**Background and Aims:**

Breast cancer is the most prevalent cancer among women worldwide, with rising trends in prevalence, incidence, and mortality rates. Genetic testing for BRCA1 and BRCA2 variants plays a pivotal role in assessing risk and enabling early detection. Despite its importance, participation in breast cancer screening programs, including genetic testing, remains notably low in Iran. This study aimed to assess Iranian women's intention to undergo predictive genetic testing for breast cancer and identify associated factors to inform policymakers and intervention strategies.

**Methods:**

A cross‐sectional online survey was conducted from July to August 2021 among 1041 Iranian women aged 30 and above. A validated questionnaire assessed demographics, psychological factors, personal and family cancer history, knowledge of breast cancer genetics, attitudes toward genetic testing, and testing intentions. Statistical analyses included Chi‐square tests, Fisher's Exact Test, and logistic regression to identify predictors of testing intention.

**Results:**

Most participants (89.5%) expressed willingness to undergo genetic testing if it were free. Higher knowledge of breast cancer genetics (OR = 1.59, *p* = 0.038) and positive attitudes toward testing (OR = 1.59, *p* = 0.042) were significant predictors of intention. Married women and those with a history of breast cancer screening were more likely to have undergone testing (*p* = 0.03 and *p* = 0.02, respectively). However, a family history of cancer and perceived risk showed no significant association with testing intentions.

**Conclusion:**

Despite limited knowledge, Iranian women demonstrated a strong interest in genetic testing for breast cancer. Educational interventions and policy measures to reduce financial barriers could enhance participation rates. Tailored programs addressing psychological concerns and improving genetic literacy are recommended to support informed decision‐making and early detection efforts.

## 1 Introduction

In 157 countries, breast cancer is the most common cancer in women [1]. There are several reports on the burden of this disease. According to the Global Burden of Diseases reports, the prevalence (8,689,255 to 20,323,179; 328.16 to 516.87 per 100,000 population), the incidence of breast cancer (865,881 to 2,082,737; 32.70 to 52.96 per 100,000 population) and death due to it (350,577 to 660,925; 13.23 to 16.80) have increased globally. This disease has resulted in 20.25 million years of life lost in 2021 (7.14% of years lost due to disability and 92.86% due to premature death) [2]. According to the statistics of the World Health Organization, in 2022, 2.3 million women will be diagnosed with breast cancer and it will be the cause of 670,000 deaths globally [1]. Physical and psychosocial functioning, family life, couple relationships, and working ability of patients are strongly affected by the disease symptoms and side effects of treatments and have a significant negative impact on their quality of life [3]. Based on a meta‐regression analysis, the patient's quality of life showed a reduction: 64.72 (EORTC QLQ‐C30), 84.39 (FACT‐B), 66.33 (QLQ‐BR23), and 57.23 (SF‐36) [4].

Screening for breast cancer is an effective measure to potentially improve the outcome and survival, as it enables early detection [5]. Breast cancer screening methods are mainly divided into two groups: breast palpation (clinical breast examination and breast self‐examination) and breast imaging techniques (mammography, ultrasonography, magnetic resonance imaging (MRI), and digital breast tomosynthesis (DBT)) [6]. Several factors such as gene variants, family and personal history of high‐risk lesions, breast density, chest radiation at a young age, and race/ethnicity are associated with breast cancer [7]. Genetic inheritance and family history play a key role in the development of this disease [8]. 5%–10% of all breast tumors develop due to deleterious variants in the breast cancer susceptibility genes, with approximately 30% attributable to pathogenic variants in BRCA1 and BRCA2, and others such as TP53, CHEK2, and PALB2 [9]. Genetic testing is, therefore, a valuable tool for cancer risk assessment, screening, and treatment planning [10]. Until recently, genetic testing was mainly prescribed for patients with a strong family history of cancer and included a limited number of genes associated with cancer and the syndromes. With the advent of cost‐effective sequencing methods, multigene susceptibility testing has become an established part of medical practice [11].

Although strong evidence has been provided on the impact of screening programs on reducing breast cancer mortality [12], there are still challenges in getting the population to participate in such programs. The participation rate in breast cancer screening is estimated at 16%–90% [13]. Considerable heterogeneity in the participation rate of Iranian women in breast cancer screening programs has been reported (3.8% to 16.8%**)** [14]. In Jafari et al.'s study, the acceptance rate of mammography in urban and rural areas was reported as 34.95% and 8.75%, respectively [15]. Meanwhile, according to the GBD^1^ reports in 2021, the mortality rate in Iranian women due to breast cancer (58.70 per 100,000 population) was higher than the global average (52.96 per 100,000 population) [2]. High participation is essential for the success of screening programs. Breast cancer screening aims to detect asymptomatic disease in apparently healthy women; however, due to the absence of noticeable symptoms, encouraging healthy women to participate in screening can be challenging [16]. Hajizadeh et al. have mentioned factors affecting the participation rate of Iranian women in screening programs. These range from fear, a lack of training programs, access to information and no awareness, cultural barriers to religious beliefs [17]. Omidi et al. have stated that in all the studies reviewed by them, educational interventions have led to the improvement of participants' knowledge, attitude, and practice in using breast cancer screening programs [14].

Knowledge is an important variable that influences behavior [18]. Negative attitudes and poor knowledge are defined as barriers to screening methods that have led to the failure of policymakers to introduce screening methods [19]. Not paying attention to the risk factors and screening methods involved, leads to late detection which increases the risk of mortality [20]. Genetic screening tests for breast cancer are a new medical technology that is not covered by the insurance system in Iran. The tests are too expensive. In this study, we aimed to assess women's intention to undergo genetic testing, allowing us to estimate the number of individuals who would ultimately participate in screening. These findings can help policymakers allocate financial resources for governmental support and contribute to designing more effective population‐wide screening programs in the future.

## 2 Methods

This cross‐sectional study focused on the intention to undergo genetic screening for breast cancer and compared it among women based on different underlying characteristics, as well as their levels of knowledge and attitudes toward the test. The study was conducted through an online survey from July 4 to August 30, 2021, in a female sample from the general population. A convenience sampling method was used. A self‐administered questionnaire based on previous studies was developed. An expert panel comprised of two health economists, two geneticists, and an oncologist confirmed the content validity of the questionnaire. Based on experts' opinions, the Scale‐Level Content Validity Index (S‐CVI) for the developed questionnaire was 0.96, which confirms its validity, given that it exceeds the recommended threshold of 0.79. According to the recommendations of Denise F. Polit et al., items with an I‐CVI of 0.78 or higher, assessed by three or more experts, are considered to have good content validity [21]. The S‐CVI was calculated as follows [22]:

$$S-\mathrm{CVI}\;\left(\mathrm{Scale}-\mathrm{level}\mathrm{Content}\mathrm{Validity}\mathrm{Index}\right)=\frac{\sum \left(I-\text{CVI}\;\mathrm{scores}\right)}{\text{Number of items}}$$

I‐CVI scores: (agreed item)/(number of expert)

To assess the reliability of the validated questionnaires, they were distributed online using a convenience sampling method among women over 30 years old. After 30 completed responses were collected, the reliability of the questionnaire was evaluated by calculating Cronbach's alpha coefficient. There are different recommendations regarding the acceptable value of Cronbach's alpha, but the threshold for acceptability is generally 0.7 [23]. For the questionnaire developed in this study, Cronbach's alpha was calculated as 0.74, indicating an acceptable level of reliability for confirming the instrument's validity.

The questionnaire was organized into seven sections, which included:

- **Introduction:** The research objectives and participation conditions were explained in detail in the introduction section of the questionnaire. As a result, the respondents were fully informed about the study's objectives, the voluntary and free nature of their participation, the researchers' commitment to maintaining confidentiality, the absence of any obligation to participate, and other relevant aspects.
- **Demographics** include age, education, occupation, income, health insurance coverage status, marital status, number of children, having/not having at least one girl.
- **Psychological parameters regarding breast cancer:** worrying about breast cancer and estimating the probability of its occurrence were categorized as psychological factors based on the previous studies [24, 25].
- **Personal and family history (FH) of cancer:** By asking Yes/No (score 1 for Yes and score 0 for No) questions, the history of breast and ovarian cancer in the respondent and blood relatives was obtained to determine the level of risk of breast cancer. Based on the obtained score, women were placed in quartiles of breast cancer risk [26].
- **Knowledge about breast cancer genetics:** The validated version of the Breast Cancer Genetic Counseling Knowledge Questionnaire (BGKQ) [27], includes 10 questions with true or false answers (true=1, false=0), was used. Based on their scores, women were classified into two groups: low and high levels of knowledge regarding breast cancer genetics.
- **Attitudes about genetic screening tests:** Pros and cons of breast screening genetic tests were assessed by nine statements with a 3‐point Likert scale as “Agree,” “Disagree,” or “Do not know” was defined based on previous studies [28, 29].
- **The intention of genetic screening tests:** The respondent's intention to participate in genetic breast cancer testing if it was free asked to estimate their participation rate. Respondents could choose “Yes,” “Not sure,” or “No.”

The study commenced after obtaining ethical approval for biomedical research from the Ethics Committee of Iran University of Medical Sciences under the code IUMS/SHMIS‐98‐4‐37‐16709. Due to the COVID‐19 pandemic and the resulting social distancing measures, in‐person data collection was not feasible. As a result, the questionnaire was developed using Google Forms and distributed through multiple digital platforms, including Telegram, Instagram, WhatsApp, and email. A convenience sampling approach was employed, whereby participants self‐selected into the study by voluntarily responding to the online survey. The inclusion criteria were identifying as female and expressing willingness to participate. The survey link was disseminated via public and semi‐public online communities. It remained open while responses were being received and was deactivated once the response rate plateaued. The collected data were entered into Microsoft Excel 2019 and then transferred to SPSS (version 23) for descriptive statistics and analysis. In addition to calculating and reporting frequencies and percentages, Categorical Data Analysis methods—including Chi‐Square, Fisher's Exact Test, Linear‐by‐Linear Association, and Chi‐Square with Monte Carlo simulation—were employed to analyze subgroups across different variables. The application of the Chi‐Square test, first introduced in 1900 by Karl Pearson for testing the goodness of fit of frequency curves, was later (in 1904) extended to contingency tables to test the independence of rows and columns. Since then, Pearson's family of Chi‐Square tests has become one of the most commonly used sets of statistical analyses in social sciences [30].

This study primarily used the Chi‐square test for subgroup comparisons, except in cases where modified versions were statistically required. Specifically, Fisher's Exact Test was applied when at least one cell in the contingency table had a frequency of less than 5 [31, 32]. For tables with numerous cells (e.g., 3 × 4 tables), the Monte Carlo simulation was employed in the Chi‐Square test to enhance efficiency [33]. Additionally, when the dependent variable was ordinal, results were interpreted based on the Linear‐by‐Linear Association test [34]. The significance level (α) was set at 0.05 for all statistical tests.

Logistic regression was used to determine the factors influencing Iranian women's intention to undergo genetic testing for breast cancer. In this model, all independent variables listed in Table 1 were included.

**Table 1:** Association of demographic, psychosocial, and knowledge factors with genetic testing for breast cancer.

Variables	Sub‐groups	Frequency, number (%)	Have you had a history of genetic testing for breast cancer?		Test	*p*‐value	Do you intend to do breast cancer genetic testing?			Test	*p*‐value
			No	Yes			No	Not sure	Yes		
Age	< 30	233 (22.4)	231 (99.1)	2 (0.9)		0.06	10 (4.3)	13 (5.6)	210 (90.1)	χ²	0.66
	≥ 30	808 (77.6)	782 (96.8)	26 (3.2)	FET*		29 (3.6)	57 (7.1)	722 (89.4)		
Education	Undergraduate	274 (26.3)	257 (93.8)	17 (6.2)	χ²**	*p* < 0.001	7 (2.6)	13 (4.7)	254 (92.7)	χ²	0.14
	Graduate	767 (73.7)	756 (98.6)	11 (1.4)			32 (4.2)	57 (7.4)	678 (88.4)		
Educated in Health‐Related Fields	Yes	432 (41.5)	424 (98.1)	8 (1.9)	χ²	0.18	18 (3.0)	46 (7.6)	545 (89.5)	χ²	0.14
	No	609 (58.5)	589 (96.7)	20 (3.3)			21 (4.9)	54 (5.6)	387 (89.6)		
Employment	Yes	523 (50.2)	515 (98.5)	8 (1.5)	χ²	0.02	21 (4.0)	42 (8.0)	460 (88.0)	χ²	0.21
	No	518 (49.8)	498 (96.1)	20 (3.9)			18 (3.5)	28 (5.4)	472 (91.1)		
Health insurance	Yes	916 (88.0)	891 (97.3)	25 (2.7)	χ²	0.56	7 (5.6)	12 (9.6)	106 (84.8)	χ²	0.18
	No	125 (12.0)	122 (97.6)	3 (2.4)			32 (3.5)	58 (6.3)	826 (90.2)		
Marital status	Single	263 (25.3)	261 (99.2)	2 (0.8)	χ²	0.03	10 (3.8)	11 (4.2)	242 (92.0)	χ²	0.17
	Married	778 (74.7)	752 (96.7)	26 (3.3)			29 (3.7)	59 (7.6)	690 (88.7)		
Having a child	Yes	618 (59.4)	595 (96.3)	23 (3.7)	χ²	0.02	21 (3.4)	43 (7.0)	554 (89.6)	χ²	0.73
	No	423 (40.6)	418 (98.8)	5 (1.2)			18 (4.3)	27 (6.4)	378 (89.4)		
Having girl	Yes	476 (45.7)	460 (96.6)	16 (3.4)	χ²	0.25	18 (3.8)	33 (6.9)	425 (89.3)	χ²	0.97
	No	565 (54.3)	553 (97.9)	12 (2.1)			21 (3.7)	37 (6.5)	507 (89.7)		
History of BC screening	Yes	308 (29.6)	294 (95.5)	14 (4.5)	χ²	0.02	11 (3.6)	20 (6.5)	277 (89.9)	χ²	0.96
	No	733 (70.4)	719 (98.1)	14 (1.9)			28 (3.8)	50 (6.8)	655 (89.4)		
History of cancer	No	1014 (97.4)	988 (97.4)	26 (2.6)	χ² (Monte Carlo)	0.11	39 (3.8)	69 (6.8)	906 (89.3)	χ² (Monte Carlo)	0.88
	Breast cancer	12 (1.2)	11 (91.7)	1 (8.3)			0 (0.0)	0 (0.0)	12 (100.0)		
	Ovarian cancer	4 (0.4)	3 (75.0)	1 (25.0)			0 (0.0)	0 (0.0)	4 (100.0)		
	Other cancers	11 (1.1)	11 (100.0)	0 (0.0)			1 (9.1)	0 (0.0)	10 (90.9)		
Family history of breast cancer	No	687 (66.0)	673 (98.0)	14 (2.0)	χ² (Monte Carlo)	0.11	28 (4.1)	46 (6.7)	613 (89.2)	χ² (Monte Carlo)	0.67
	First‐degree relative	94 (9.0)	87 (92.6)	7 (7.4)			2 (2.1)	5 (5.3)	87 (92.6)		
	Second‐degree relative	203 (19.5)	197 (97.0)	6 (3.0)			7 (3.4)	12 (5.9)	184 (90.6)		
	First and Second degree‐relative	3 (0.3)	3 (100.0)	0 (0.0)			0 (0.0)	0 (0.0)	3 (100.0)		
	Uninformed	54 (5.2)	53 (98.1)	1 (1.9)			2 (3.7)	7 (13.0)	45 (83.3)		
Family history of ovarian cancer	No	821 (78.9)	799 (97.3)	22 (2.7)	χ² (Monte Carlo)	0.41	32 (3.9)	53 (6.5)	736 (89.6)	χ² (Monte Carlo)	0.28
	First‐degree relative	36 (3.5)	35 (97.2)	1 (2.8)			3 (8.3)	1 (2.8)	32 (88.9)		
	Second‐degree relative	83 (8.0)	79 (95.2)	4 (4.8)			3 (3.6)	9 (10.8)	71 (85.5)		
	Uninformed	101 (9.7)	100 (99.0)	1 (1.0)			1 (1.0)	7 (6.9)	93 (92.1)		
Risk of BC	Low Risk	700 (67.2)	678 (96.9)	22 (3.1)	χ²	0.23	22 (3.1)	49 (7.0)	629 (89.9)	χ²	0.31
	High Risk	341 (32.8)	335 (98.2)	6 (1.8)			17 (5.0)	21 (6.2)	303 (88.9)		
Knowledge of BC genetics	Low Knowledge	587 (56.4)	569 (96.9)	18 (3.1)	χ²	0.44	25 (4.3)	49 (8.3)	513 (87.4)	χ²	0.03
	high Knowledge	454 (43.6)	444 (97.8)	10 (2.2)			14 (3.1)	21 (4.6)	419 (92.3)		
Attitude towards BC genetics	Low agreement	231 (22.2)	225 (97.4)	6 (2.6)	χ²	0.57	19 (8.2)	16 (6.9)	196 (84.8)	χ²	*p* < 0.001
	High agreement	810 (77.8)	788 (97.3)	22 (2.7)			20 (2.5)	54 (6.7)	736 (90.9)		
How do you assess the probability of getting breast cancer?	Low	590 (56.7)	577 (97.8)	13 (2.2)	χ² (LLA)	0.17	25 (4.2)	41 (6.9)	524 (88.8)	χ² (LLA)	0.74
	Moderate	415 (39.9)	403 (97.1)	12 (2.9)			11 (2.7)	28 (6.7)	376 (90.6)		
	High	24 (2.3)	22 (91.7)	2 (8.3)			3 (12.5)	1 (4.2)	20 (83.3)		
How worried are you about getting breast cancer?	Low	481 (46.2)	474 (98.5)	7 (1.5)	χ² (LLA***)	0.05	23 (4.8)	39 (8.1)	419 (87.1)	χ² (LLA)	0.02
	Moderate	87 (8.4)	83 (95.4)	4 (4.6)			4 (4.6)	4 (4.6)	79 (90.8)		
	High	461 (44.3)	445 (96.5)	16 (3.5)			12 (2.6)	27 (5.9)	422 (91.5)		
Total		1041	1013 (97.3)	28 (2.7)		‐‐‐	39 (3.7)	70 (6.7)	932 (82.5)		‐‐‐

* Fisher's exact test.

** Chi‐square test.

*** Linear by linear association test.

All authors have read and approved the final version of the manuscript (Soheila Damiri) had full access to all of the data in this study and take complete responsibility for the integrity of the data and the accuracy of the data analysis.

## 3 Results

3845 individuals viewed the questionnaire, and 1041 completed it online (response rate = 27.07%). The majority of participants were over 30 years old (77.6%), university‐educated (73.7%), employed (50.2%), and married (74.7%). Additionally, 59.4% had at least one child, and 45.7% had a daughter. Most participants had health insurance (88.0%). Regarding medical history, 70.4% had never undergone breast cancer screening, and 97.4% had no personal history of cancer. Among those with a history of cancer (2.6%), 1.2% had breast cancer, 0.4% had ovarian cancer, and 1.1% reported other cancers. A family history of breast cancer was reported by 28.8% of participants, while 11.1% had a family history of ovarian cancer. When assessing breast cancer risk perception, 67.2% of participants considered themselves at low risk, while 32.8% perceived a high risk. Additionally, 56.4% had low knowledge of breast cancer genetics, and 22.2% had a low level of agreement with genetic testing. Among all participants, only 2.7% had previously undergone genetic testing for breast cancer. Regarding future intentions, 89.5% expressed willingness to undergo genetic testing, 6.7% were unsure, and 3.7% stated they had no intention to do so (Table 1).

Education level (*p* < 0.001), and employment status (*p* = 0.02) were significantly associated with previous genetic testing, but no significant association was found between employment and future testing intention (*p* = 0.21). Marital status was significantly associated with prior genetic testing (*p* = 0.03), with married women being more likely to have undergone testing while having children also showed a significant association with past testing behavior (*p* = 0.02) but not with future intentions (*p* = 0.73). History of breast cancer screening was a predictor of prior genetic testing (*p* = 0.02), and family history of breast or ovarian cancer did not show any significant associations with past or future testing intentions (*p* > 0.05). Perceived breast cancer risk was not significantly linked to genetic testing behavior (*p* = 0.23 for past testing, *p* = 0.31 for future intention). However, knowledge of breast cancer genetics was significantly associated with undergoing genetic testing (*p* = 0.03), suggesting that increased awareness may lead to a higher willingness for testing. Attitudes toward breast cancer genetics were significantly associated with genetic testing intention (*p* < 0.001). Additionally, higher levels of worry about breast cancer were significantly linked to an increased likelihood of intending to undergo genetic testing (*p* = 0.02). In contrast, age did not show a significant association with genetic testing behavior (*p* = 0.66 for intention). Overall, the findings suggest that education, employment, marital status, prior breast cancer screening, and attitude toward genetic testing were key predictors of genetic testing behavior, while knowledge of breast cancer genetics and worry about breast cancer played significant roles in shaping future testing intentions. Conversely, age, family history, and perceived risk did not have significant associations with genetic testing behavior (Table 1).

Table 2 presents the distribution of responses to knowledge questions about breast cancer genetics. For instance, 28.8% correctly recognized that a father can pass down a breast cancer gene to his daughters, while 48.5% were uncertain. Only 5.7% correctly identified that breast cancer genes do not cause half of all breast cancers, with 54.9% believing the false statement. Additionally, 59.6% knew that women without a breast cancer gene can still develop the disease. Understanding of hereditary risks was mixed, with 54.0% recognizing the link between breast and ovarian cancer inheritance, while misconceptions remained prevalent.

**Table 2:** The distribution of answers to the knowledge questions.

Items	Don't know	False	Correct
A father can pass down a breast cancer gene to his daughters	48.5	22.7	28.8
Breast cancer genes cause about one‐half of all breast cancers	39.4	54.9	5.7
A woman who does not have a breast cancer gene can still get breast cancer	35.6	4.8	59.6
Everybody has the BC gene	56.8	5.5	37.8
All women who have a breast cancer gene will get breast cancer	50.7	21.6	27.7
If breast cancer is inherited in a family, they are at risk of ovarian cancer, too	38.8	9.4	51.8
A woman who has a sister with a breast cancer gene has a 50% chance of having the gene herself	30.5	15.6	54.0
A woman who has an altered breast or ovarian cancer has a 50% chance (1 in 2) of passing an altered breast or ovarian cancer gene to each of her children	36.4	10.5	53.1
Even if a woman does not have an altered gene, her children can still get it from their grandmother (their mother's mother)	33.8	4.4	61.8
A woman who has had her breasts removed can still get cancer	38.4	44.2	17.4
Total	40.9	19.4	39.8

Most respondents (84.1%) agreed that genetic tests provide personal risk information, and 91.9% felt knowing their gene status would encourage more frequent self‐examinations. Additionally, 89.3% believed gene awareness would enhance their sense of personal control. Concerns about confidentiality were lower, with only 17.1% expressing worry. However, 45.2% lacked trust in modern medicine, and 25.9% wrongly believed nothing could prevent cancer. These findings highlight strong support for genetic testing but indicate lingering concerns about trust and confidentiality (Table 3).

**Table 3:** The distribution of answers to attitude questions.

Items	No idea	Not agree	Agree
Genetic tests can help to inform about the personal risk of breast cancer.	13.4	2.6	84.1
Knowing whether or not I carry the gene would increase my sense of personal control.	8.8	1.8	89.3
If I were found to carry the gene, it would help my daughter(s) or sister(s) decide whether to undergo genetic testing.	13.4	1.6	85.0
If I were found to carry the gene, I could make childbearing decisions.	20.7	7.4	71.9
Knowing that I carry the gene would motivate me to perform breast self‐examination more frequently.	7.1	1.0	91.9
If I were found to carry the gene, I would be worried about confidentiality loss.	15.8	67.1	17.1
I do not trust modern medicine.	39.3	15.5	45.2
I am concerned about the effect it would have on my family.	35.0	38.4	26.6
I believe that there is nothing that can be done to prevent getting cancer.	20.4	53.7	25.9
Total	21.0	59.7	19.3

Based on binary regression in addition to knowledge and attitude towards genetic screening tests, the probability of breast cancer gene variants is significantly associated with doing genetic screening tests for breast cancer (Table 4).

**Table 4:** Logistic regression analysis of factors associated with breast cancer genetic screening intention.

Predictor variables	B	*S.E.*	*p*‐value	Exp(B)
Probability of breast cancer gene variant	0.39	0.15	0.011	1.471
Attitude	0.46	0.28	0.042	1.586
Knowledge	0.46	0.22	0.038	1.590
Constant	1.38	0.20	< 0.001	3.970

## 4 Discussion

This study investigated Iranian women's intentions to undergo BRCA genetic testing for breast cancer susceptibility. Insights into these intentions are critical for guiding public health strategies and informing the design of financial support and insurance coverage mechanisms. The results showed that over 89% of participants intended to pursue genetic testing. Binary logistic regression analysis identified knowledge, attitudes toward genetic screening, and the perceived probability of carrying a BRCA gene variant as significant predictors of this intention. Despite the clinical value of genetic screening in early cancer prevention, its adoption in Iran remains limited, primarily due to high costs and lack of insurance reimbursement. The findings illustrate a nuanced understanding of Iranian women's attitudes towards genetic screening. Despite limited knowledge of breast cancer genetics, participants exhibited a strong positive attitude and a high intention to undergo genetic testing—particularly among those identified as high‐risk. This trend is consistent with international evidence. For example, a study by O. Ngene et al. reported that while 84% of Nigerian women demonstrated low knowledge of breast cancer genetics, over 87% expressed willingness to undergo genetic testing. In the Nigerian context, factors influencing this willingness included religious affiliation, perceived personal risk when a close relative carried a genetic variant, and access to healthcare services [35]. A similar disconnect between knowledge and intention was observed in this study. Although participants demonstrated a limited understanding of breast cancer genetics, there was a significant interest in undergoing genetic screening. Religious affiliation was not explicitly analyzed, as more than 90% of Iranian women identify as Muslim, leading to a relatively homogeneous study sample. The high intention to pursue genetic testing highlights a valuable opportunity for targeted educational interventions. Enhancing awareness of breast cancer genetics and the benefits of screening may contribute to increased uptake of testing services. The expressed willingness to engage in genetic screening, even with limited understanding, provides a strong foundation for the implementation of broader breast cancer prevention strategies. Nonetheless, this also underscores the necessity of structured educational programs to support informed decision‐making regarding genetic testing and its implications. The differing results between this study and the research by Terui‐Kohbata et al. suggest that knowledge, attitudes, and intentions regarding hereditary breast cancer and genetic testing vary across cultural and demographic contexts. Terui‐Kohbata et al. examined Japanese female university students aged 20–30 and reported high levels of knowledge and strong interest in genetic testing. Interestingly, women at higher risk showed less interest in testing (74.5%) than those at lower risk (92%) [36]. In contrast, Iranian women in the present study exhibited a strongly held intention to undergo genetic testing, despite possessing limited knowledge. These differences may reflect variations in age, life stage, educational experiences, and cultural norms. For instance, the broader age range and diverse backgrounds of Iranian women may have contributed to more immediate concerns regarding health risks. On the other hand, the younger, more homogeneous Japanese sample may have viewed genetic testing through a more future‐oriented lens. Additionally, contrasting perceptions of personal risk and cultural attitudes toward family planning could account for differences in testing intentions. These findings underscore the importance of designing genetic counseling and educational programs that are responsive to the cultural and demographic characteristics of the population to support informed decision‐making.

Hurtado‐de‐Mendoza et al. conducted a study examining the knowledge of breast cancer genetics among Black women with BRCA variants, revealing that approximately 98% of respondents had breast cancer, with an average age of 51 years and only 42% being married. The findings indicated moderate levels of understanding regarding breast genetics, and multivariable regression analysis identified high knowledge and self‐efficacy as significant factors influencing the use of genetic counseling and testing [37]. This underscores the critical need for targeted educational programs to enhance awareness and encourage proactive health measures, particularly in communities with a higher prevalence of BRCA variants. By fostering knowledge and confidence, these initiatives can empower black women to engage more fully with genetic counseling and testing services, ultimately improving health outcomes in this vulnerable population.

While numerous studies advocate for enhanced education about breast cancer genetics for women [35, 37], research by Brédart et al. reveals a concerning paradox: women with BRCA variants who possess high levels of genetic knowledge often experience significant psychological distress [38]. This heightened anxiety may stem from an acute awareness of their cancer risks, the cognitive burden of complex genetic information, and an intensified sense of vulnerability. Consequently, healthcare providers must adopt a balanced approach to genetic education, ensuring that information is tailored to individual needs and accompanied by robust psychological support. By framing genetic knowledge as a means of empowerment rather than a source of anxiety, and by providing ongoing emotional care, healthcare professionals can help mitigate distress while promoting informed decision‐making among women facing the challenges of BRCA variants.

This study has several notable limitations. First, the reliance on convenience sampling may limit the generalizability of findings, as it likely underrepresented low‐income and rural populations with limited internet access. Second, self‐reported data are susceptible to social desirability bias, as participants may have over‐reported their intention to undergo genetic testing or provided socially acceptable responses regarding attitudes toward breast cancer screening. Third, while financial barriers were acknowledged, the study did not quantitatively assess how the cost might affect testing uptake despite high expressed interest (89.5%). Fourth, the predominantly urban sample (notably from Tehran) may not reflect cultural and socioeconomic diversity across Iran. Fifth, as a cross‐sectional study, it could not address psychological sequelae (e.g., posttest anxiety) or longitudinal behavioral outcomes, which are vital for program sustainability. These limitations highlight the need for future research with geographically diverse samples, mixed‐methods approaches, and longitudinal tracking of testing behaviors and their psychosocial impacts. Finally, because the questionnaire was disseminated through public and semi‐public social media platforms (e.g., Telegram, WhatsApp), the total number of individuals who received or accessed the link could not be accurately ascertained. As a result, calculating a traditional response rate was unfeasible. This limitation is frequently encountered in online surveys that utilize convenience sampling techniques.

## 5 Conclusion

This study investigated the intentions of Iranian women regarding breast cancer genetic testing and found a high intention of testing among participants. However, the actual uptake may vary due to psychological factors and a narrow focus on breast cancer cases. To maximize participation in breast cancer screening programs, individuals must first undergo evidence‐based risk assessments and receive personalized risk awareness education. Implementing targeted training initiatives and preventive interventions through evidence‐informed policies can address existing gaps in care, ultimately reducing the incidence of advanced‐stage breast cancer. A comprehensive risk assessment system should actively communicate personalized risk data to empower preventive decision‐making. Policymakers must prioritize two key approaches [1]: developing educational campaigns to facilitate informed decision‐making, and [2] implementing preventive interventions to promote proactive health behaviors among at‐risk women. Furthermore, leveraging technological advancements—particularly through social media platforms—can significantly enhance public awareness and participation in screening programs.

## Author Contributions

**Zahra Meshkani:** conceptualization, methodology, data curation, supervision, validation, writing – original draft, writing – review and editing. **Soheila Damiri:** methodology, software, formal analysis, writing – original draft, writing – review and editing. **Najmeh Moradi:** conceptualization, writing – original draft. **Mohadeseh Motamed‐Jahromi:** investigation, validation, data curation. **Ali Aboutorabi:** conceptualization, methodology, investigation, writing – original draft.

## Ethics Statement

This study was part of a Ph.D. thesis that is supported by Iran University of Medical Sciences (grant No: IUMS/SHMIS‐98‐4‐37‐16709, and Ethical Code.IR.IUMS.REC.1398.1051).

## Consent

This was not a clinical study, although all participants in the study answered the questions voluntarily. The authors confirmed that informed consent was obtained from all subjects.

The authors confirm that all methods were performed following the relevant guidelines and regulations e.g., the Declaration of Helsinki.

## Conflicts of Interest

The authors declare that there is no conflict of interest regarding the publication of this article. This study did not receive any specific funding from public, commercial, or non‐profit funding agencies. Iran University of Medical Sciences didn't have any role in study design; collection, analysis, and interpretation of data; writing of the report; and the decision to submit the report for publication.

## Transparency Statement

The lead author Soheila Damiri affirms that this manuscript is an honest, accurate, and transparent account of the study being reported; that no important aspects of the study have been omitted; and that any discrepancies from the study as planned (and, if relevant, registered) have been explained.

## Data Availability

The data that support the findings of this study are available from the corresponding author upon reasonable request.

## Endnotes

1 Global Burden of Disease.
